# Expression and prognostic impact of the protein tyrosine phosphatases PRL-1, PRL-2, and PRL-3 in breast cancer

**DOI:** 10.1038/sj.bjc.6603261

**Published:** 2006-07-11

**Authors:** I Radke, M Götte, C Kersting, B Mattsson, L Kiesel, P Wülfing

**Affiliations:** 1 Department of Obstetrics and Gynaecology, University of Münster, Albert-Schweitzer-Str. 33, D-48149 Münster, Germany; 2 Gerhard-Domagk-Institute of Pathology, University of Münster, Domagkstr. 17, D-48149 Münster, Germany

**Keywords:** immunohistochemistry, breast cancer, PRL-3, PTP4A3, prognostic factor

## Abstract

The aim of this study was to investigate the expression of the protein tyrosine phosphatases (PTP) PRL-1, PRL-2, and PRL-3 in human breast cancer and to evaluate its clinical and prognostic significance. PRL-PTP mRNA expression was examined in malignant (*n*=7) and nonmalignant (*n*=7) cryoconserved breast tissue samples as well as in eight breast cancer cell lines by RT–PCR. Furthermore, protein expression of PRL-3 was analysed semiquantitatively by immunohistochemistry in ductal breast carcinoma *in situ* (*n*=135) and invasive breast cancer (*n*=147) by use of tissue microarray technology (TMA). In 24 lymph node-positive patients we selected the corresponding lymph node metastases for analysis of PRL-3 expression, and a validation set (*n*=99) of invasive breast cancer samples was examined. Staining results were correlated with clinicopathological parameters and long-term follow-up. PRL-3 mRNA expression was significantly higher in malignant compared to benign breast tissue. For PRL-1 and PRL-2 expression no significant differences were observed. Staining of TMAs showed PRL-3 expression in 85.9% ductal carcinoma *in situ* and 75.5% invasive breast carcinomas. Analysis of survival parameters revealed a shorter disease-free survival (DFS) in patients with PRL-3-positive carcinomas, and in particular a significantly shorter DFS in nodal-positive patients with PRL-3 overexpressing tumours as compared to PRL-3-negative breast carcinomas (66±7 months (95% CI, 52–80) *vs* 97±9 months (95% CI, 79–115); *P*=0.032). Moreover, we found a more frequent expression of PRL-3 in lymph node metastases as compared to the primary tumours (91.7 *vs* 66.7%; *P*=0.033). Our results suggest that PRL-3 might serve as a novel prognostic factor in breast cancer, which may help to predict an adverse disease outcome.

Protein tyrosine phosphatases (PTPs) play a fundamental role in regulating diverse proteins that essentially participate in every aspect of cellular physiologic and pathologic processes (Zeng *et al*, 1998). The PTP PRL-3, also known as PTP4A3, belongs to a group of three PTPs (PRL-1, PRL-2, and PRL-3), which share a 76–87% sequence identity and a unique COOH-terminal prenylation motif with a PTP-active site signature sequence (Diamond *et al*, 1994; Zeng *et al*, 1998). PRL-1 was the first to be identified, originally as an immediate early gene, the expression of which was induced in mitogen-stimulated cells and in regenerating liver, therefore named ‘protein of regenerating liver’ (PRL) (Mohn *et al*, 1991; Montagna *et al*, 1995). Overexpression of PRL-1 and PRL-2 has been found to transform mouse fibroblasts and pancreatic epithelial cell *in vitro* and to promote tumour growth in nude mice, suggesting that they might play a role in tumourigenesis (Diamond *et al*, 1994; Cates *et al*, 1996). Migration and invasion have been shown to be enhanced by PRL-3 and PRL-1 expression in Chinese hamster ovary cells and overexpression of these proteins induced metastatic tumour formation in mice (Zeng *et al*, 2003). Recent studies showed, that PRL-3 expression is associated with human ovarian cancer progression (Polato *et al*, 2005). PRL-3 is overexpressed in metastatic colorectal and gastric cancer (Miskad *et al*, 2004), whereas nonmetastatic colorectal and gastric cancer did not show PRL-3 overexpression (Bardelli *et al*, 2003; Kato *et al*, 2004). Also, PRL-3 expression in metastases of colorectal cancer (CRC) is significantly higher than in the primary tumour itself or in normal colorectal epithelia (Peng *et al*, 2004). Results of these studies suggest that an excess of PRL-3 may play a key role in the acquisition of metastatic potential of tumour cells. To date, with respect to breast cancer, there are only data on PRL-3 expression in several breast cancer cell lines available, as recently reported by Rouleau *et al* (2006).

Tumour angiogenesis is an important prerequisite of tumour growth and progression (Folkman, 1995). Among others, one of the most crucial regulators of angiogenesis is the vascular endothelial growth factor (VEGF) (reviewed in Morabito *et al*, 2004). In breast cancer, expression of VEGF is correlated with angiogenesis and seems to represent a useful prognostic marker for poor clinical outcome (Yoshiji *et al*, 1996; Obermair *et al*, 1997; de Jong *et al*, 2001). Parker *et al* (2004) reported that PRL-3 is expressed in breast tumour vasculature. In other tumour entities, evidence of a causative role of PRL-3 in tumour-related angiogenesis has been demonstrated (Bardelli *et al*, 2003). However, to date, there is little data available on expression of PRL-PTPs in breast cancer and their potential involvement in angiogenesis.

In this study, we analysed expression of PRL-PTPs in malignant and nonmalignant breast tissues and characterised several human breast cancer cell lines with respect to expression of PRL-PTPs. Furthermore, we evaluated the clinical and prognostic significance of PRL-3 expression in human breast cancer and its potential role in tumour angiogenesis.

## MATERIALS AND METHODS

### Cell lines and tissue samples

Eight human breast cancer cell lines MCF-7, HBL-100, BT-474 (a gift from C Poremba, Düsseldorf, Germany), SK-BR-3, MDA-MB-468 (B Brandt, Münster, Germany), MDA-MB-453 (A Rody, Frankfurt, Germany), DU-4475 (C Bremer, Münster, Germany), and MDA-MB-231 (CLS Cell Lines Services, Eppelheim, Germany) as well as cryoconserved malignant and nonmalignant breast tissue samples (each: *n*=7) were selected for analysis.

### RT–PCR analysis

Total cellular RNA from all cancer cell lines and breast tissues was prepared using the RNeasy™ Mini Kit together with the RNaseFree-DNase Set™ (Qiagen Inc., Valencia, CA, USA) to remove contaminating genomic DNA. Total RNA was reverse-transcribed with the Advantage™ reverse transcription (RT)-for-polymerase chain reaction (PCR) Kit (BD Clontech, Heidelberg, Germany). Briefly, 2.5 *μ*g of RNA was reverse-transcribed and amplified for 30 cycles (PRL-1), 26 cycles (PRL-2) and 28 cycles (PRL-3) with an annealing temperature of 60°C. Primer sequences for PRL-1 were 5′-TACTGCTCCACCAAGAAGCC-3′ (forward) and 5′-AGGTTTACCCCATCCAGGTC-3′ (reverse); for PRL-2 5′-ACTTTCCCCATCACACTCAC-3′ (forward) and 5′-CCTCTAAATGGCACAATCAAG-3′ (reverse) and for PRL-3 5′-GGGACTTCTCAGGTCGTGTC-3′ (forward) and 5′-AGCCCCGTACTTCTTCAGGT-3′ (reverse). The housekeeping gene *β*-actin was used as an internal control. Polymerase chain reaction products were subjected to gel electrophoresis on 1.5% agarose gels under standard conditions (Samrook *et al*, 1989). After staining with ethidium bromide, DNA bands were photographed under UV illumination using a BioDoc analysing system (Biometra, Göttingen, Germany). Semiquantitative analysis was carried out using ImageJ software version 1.34 (National Institute of Health, MD, USA). The relative amounts of PRL-PTP mRNA expression were normalised to *β*-actin expression. All experiments were performed in triplicate.

### Transient transfection of MCF-7 cells and Western blotting

A polyclonal rabbit-anti-PRL-3 antibody (Zymed, South San Francisco, CA, USA) was selected for immunohistochemical analysis. The peptide sequence that Zymed, Inc., selected for the antibody was specific for PRL-3 and differed from PRL-1 (only 4 a.a. out of 10 a.a. being identical) and PRL-2 (only 2 a.a. out of 10 a.a. being identical, G Hirsch, Invitrogen Ltd, personal communication). Moreover, Miskad *et al* (2004) had previously proven by Western blotting of gastric carcinoma cell extracts that the antibody is specific for PRL-3. To provide further evidence for the specificity of the antibody, we probed Western blots of MCF-7 cells overexpressing PRL-3 with the PRL-3 antiserum as follows: MCF-7 cells were plated in six-well tissue culture plates at 70% confluency and cultured as previously described (Wülfing *et al*, 2005b). After 24 h, the cells were transiently transfected with the control plasmid pcDNA3.1 (Invitrogen, Karlsruhe, Germany) or with the PTP4A3v1 human phosphatase vector (Stratagene, Heidelberg, Germany) allowing for the overexpression of PRL-3 under the control of the CMV promoter. The Effectene transfection reagent (Qiagen, Hilden, Germany) was used following the manufacturers instructions. At 48 h after transfection cells were lysed and 50 *μ*g protein/lane were subjected to SDS–PAGE on 4–15% gradient gels (Biorad, Munich, Germany) and electrotransfer to nitrocellulose membranes exactly as described (Sonntag *et al*, 2005). Immunoblotting was performed by treating nitrocellulose membranes with blocking buffer (5% skim milk in 0.1% TBS-Tween (TBST)) for 1 h at room temperature, followed by incubation with the PRL-3 antibody (1 : 1000 in TBST/5% BSA) overnight at 4°C. After washing in TBST buffer three times, the membranes were incubated for 1 h with horseradish peroxidase-conjugated anti-rabbit IgG diluted 1 : 2000 (Cell Signaling Technology, Beverly, MA, USA) in blocking buffer. The membranes were washed and treated with enhanced chemiluminescence detection reagents (Super Signal, Pierce, Bonn, Germany) for 1 min, exposed to Hyperfilm-ECL for 45 min. The chemoluminescence signal on developed films was documented using a BioDoc analyzing system (Biometra, Göttingen, Germany).

## PATIENTS

To study PRL-3 expression in preinvasive and invasive breast cancer, in this study tissue samples from 135 patients with ductal carcinoma *in situ* (DCIS) and 147 patients with invasive breast cancer, diagnosed between 1993 and 1997 at the Department of Gynaecology, University Münster, Germany, were analysed. Details on patients' characteristics have been described previously (Wülfing *et al*, 2003 and 2005a). In addition, in 24 lymph node-positive patients we selected the corresponding lymph node metastases for analysis of PRL-3 expression. For validation of obtained data a second set of specimens from 99 breast cancer patients, diagnosed between 1988 and 1990, has been selected. Follow-up of these patients was evaluated until April 2004. At time of diagnosis, mean age of the patients was 57.0 months (range: 33–82 years). Mean disease-free survival (DFS) in this subset was 102.6 months (range, 1–197 months; median, 97.0 months; s.d., 75.2 months), and mean overall survival (OS) was 113.0 months (range 5–197 months; median, 161 months; s.d., 72.3 months). For all patients informed consent was obtained prior to the study and the local ethical committee approved use of tumour tissue. Detailed clinical data regarding diagnosis, histopathological variables, treatment, and follow-up were collected and stored in a database. Routinely fixed paraffin-embedded tissue samples of patients were obtained from the archives of the Gerhard-Domagk-Institute of Pathology, University of Münster, Germany. All DCIS cases were classified according to the criteria outlined by Holland *et al* (1994) considering the nuclear grading and architectural features. Based on this classification, cases were graduated as low grade (*n*=38), intermediate grade (*n*=32), and high grade (*n*=62); three cases could not be graded. With respect to these criteria, cases were divided into ‘non-high-grade’ (low grade + intermediate grade) and ‘high grade’ DCIS. Among the invasive breast carcinomas in the first tissue microarray technology (TMA)-series (subset 1) 80 (54.4%) were ductal invasive, 33 (22.5%) lobular, 3 (2.0%) tubular, 8 (5.4%) mucinous, 4 (2.7%) medullary, and 19 (12.9%) of mixed histological differentiation. The second TMA-series (subset 2) used for validation of results consisted of 64 (64.6%) ductal invasive, six (6.1%) lobular, one (1.0%) tubular, one (1.0%) scirrhous, and 17 (17.2%) invasive breast carcinomas of mixed histological differentiation. The tissue specimens were classified according to the Tumour-Node-Metastasis classification of the International Union Against Cancer, and tumour grade was assigned based on the criteria of Elston and Ellis (2002). Table 1 summarises the distribution of Tumour-Node-Metastasis stages and histological grade in the invasive breast carcinomas in both subsets. To enable simultaneous analysis of tissues using the same reaction, tissue microarrays were used for immunohistochemical studies. Preparation of TMAs was performed as described previously (Wülfing *et al*, 2004). In brief, for each of the cases a representative tumour block was selected as donor block. Using a haematoxylin and eosin stained slide, at least three morphologically representative regions were defined for each of the preinvasive and invasive breast cancer specimens. From these regions cylindrical core tissue specimens (diameter=0.6 mm) were acquired and precisely arrayed into several recipient paraffin blocks (20 × 35 mm) using a custom-built precision instrument (Beecher Instruments, Silver Spring, MD, USA).

### Immunohistochemistry

Consecutive sections of 2–3 *μ*m were cut from the TMAs and processed for immunohistochemistry. Before PRL-3 staining, specimens were subjected to heat-induced antigen retrieval in a steamer (Type 3216, Braun, Kronberg, Germany). Immunohistochemical staining for PRL-3 was performed in a multistep semiautomated procedure (Dako-Autostainer). A polyclonal rabbit antibody (Zymed, South San Francisco, CA, USA) was used at a dilution of 1 : 100. Smooth muscle tissue from appendices known to express PRL-3 served as positive, omission of the primary antibody as negative control. After counterstaining with haematoxylin, cytoplasmatic and nuclear PRL-3 staining was scored according to the staining intensity: almost no staining (0), moderate (1), and strong staining (2). We defined samples with a moderate or strong immunostaining intensity to have an elevated PRL-3 expression and thus to be positive. Immunohistochemical staining for endothelin-A-receptor (ET_A_R) and VEGF was performed as described previously (Wülfing *et al*, 2004). Staining results were evaluated semiquantitatively in a blind fashion by two independent investigators. In case of conflicting results between the two observers, the higher score was used for statistical analysis.

### Confocal immunofluorescence microscopy

Tissue microarrays were subjected to antigen retrieval as described in the immunohistochemistry section. The sections were blocked with PBS/2% goat serum for 1 h at room temperature (RT) and incubated with rabbit-anti-PRL-3 antibody (Zymed) and mouse-anti-human CD34 antibody (BD Pharmingen, Heidelberg, Germany) diluted 1 : 100 in PBS/1% BSA for 16 h at 4°C in a humid chamber. Vascular endothelium and haematopoietic progenitor cells specifically express CD34. Following four washes with PBS, the samples were incubated with AlexaFluor 546-conjugated goat-anti-mouse IgG (Molecular Probes, Eugene OR, USA, 1 : 600) and AlexaFluor 488-conjugated donkey-anti-rabbit IgG (Molecular Probes, 1 : 600) in the dark for 1 h. One TMA was incubated with secondary antibodies only for control purposes. Slides were mounted with VectaShield (Vector Labs, Burlingame, CA, USA). Laser scanning microscopy was performed with a Leica TCS SL confocal microscope.

### Data analysis

*T*-test was used to test for differences in PRL-PTP expression. Semiquantitative analysis of staining results was performed in blind-trial fashion without knowledge of the clinical data for the corresponding case. Correlations between PRL-3 expression and clinicopathological parameters were tested for statistical significance by *χ*^2^ test using SPSS Version 11.0 for Windows. For analysis of survival data related to PRL-3 expression, Kaplan–Meier survival estimates were generated and compared by the log-rank test. Disease-free survival was calculated as the time from the date of diagnosis to the occurrence of locoregional or distant metastasis or death. Overall survival was defined as the time from diagnosis to death from breast cancer. Multivariate analysis was performed using Cox's proportional hazards regression model. *P*-values less than 0.05 were considered statistically significant.

## RESULTS

### PRL-PTP mRNA expression in breast tissues and human breast cancer cell lines

**Figure 1:**
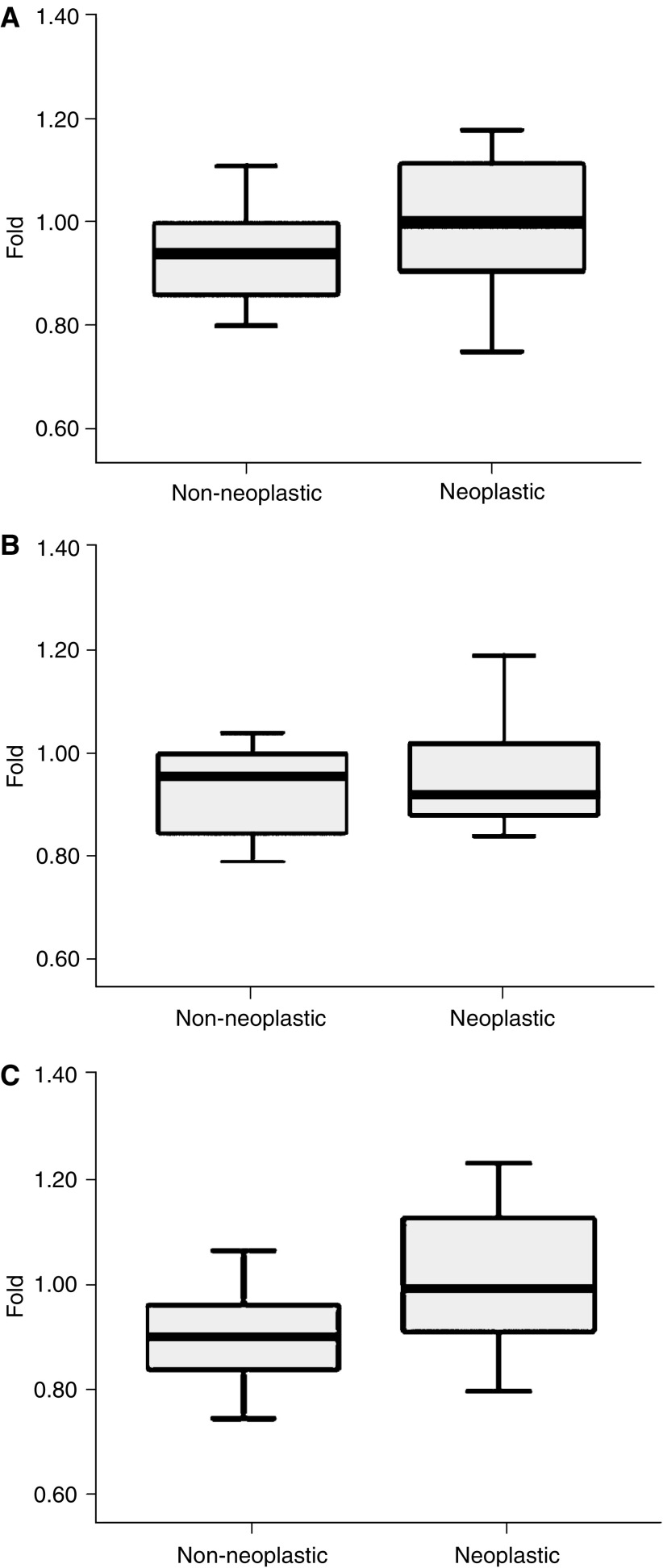
Boxplots comparing (**A**) PRL-1, (**B**) PRL-2, and (**C**) PRL-3 mRNA expression in non-neoplastic and neoplastic breast cancer specimens. Only PRL-3 showed a significantly higher expression in breast cancer as compared to normal breast tissue (*P*=0.010).

**Figure 2:**
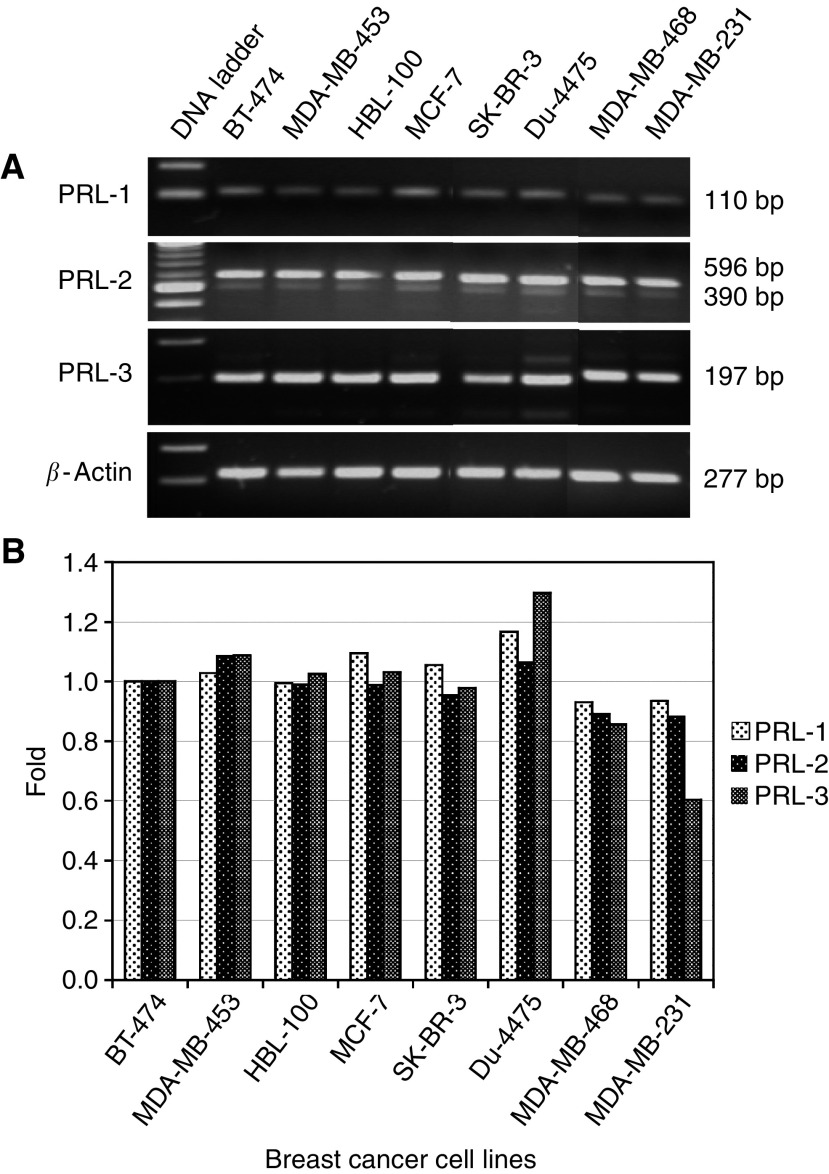
Expression of PRL-1, PRL-2, and PRL-3 mRNA in different human breast cancer cell lines. (**A**) Agarose gel after ethidium bromide staining. (**B**) Semiquantitative evaluation of PRL-1, PRL-2, and PRL-3 mRNA expression levels.

PRL-3 mRNA expression was significantly higher in neoplastic compared with non-neoplastic breast cancer tissue specimens (mean 1.015±0.156 *vs* 0.898±0.089; *P*=0.010). There was a trend to higher expression in malignant tissue but no significant difference in the expression of PRL-1 (1.071±0.288 *vs* 0.940±0.086; *P*=0.080) and no difference with respect to PRL-2 expression (0.974±0.154 *vs* 0.929±0.082; *P*=0.292) as shown in Figure 1. All human breast cancer cell lines evaluated in this study showed expression of PRL-1, PRL-2, and PRL-3 mRNA. Different expression levels of PRL-PTPs in these cell lines are shown in Figure 2.

### Western blot analysis for specificity of the PRL-3 antibody

**Figure 3:**
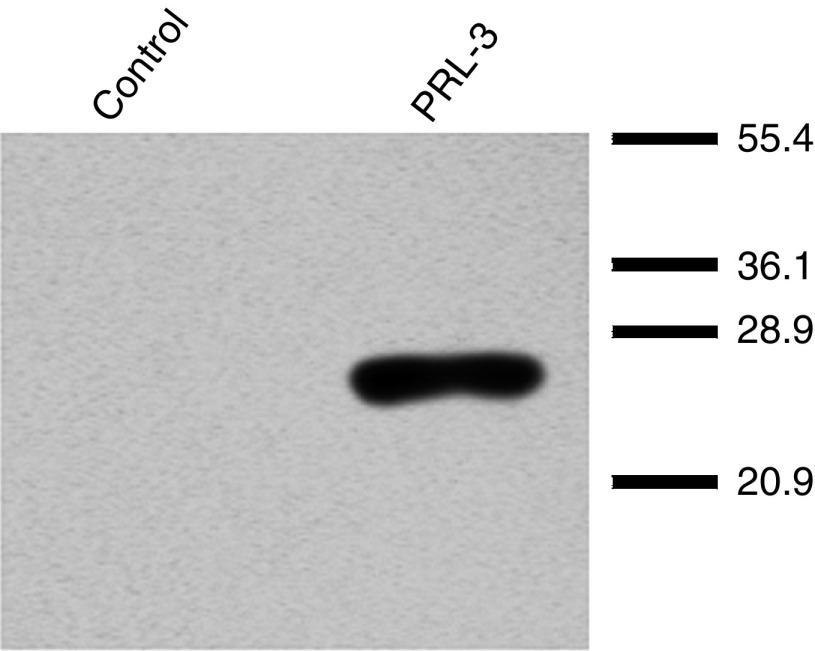
Western blot analysis for specificity of the PRL-3 antibody. A specific band of about 25 kDa was detected in PRL-3 overexpressing MCF-7 cells.

For validation of the PRL-3 antibody used for immunohistochemistry, we performed Western blot analysis of PRL-3 up regulated breast cancer cells. MCF-7 cells were transiently transfected with a control plasmid and a plasmid overexpressing PRL-3. Fifty microgram of cell lysate/lane were subjected to SDS–PAGE and Western blotting using the PRL-3 antibody. A specific band of about 25 kDa was detected in PRL-3 overexpressing MCF-7 cells (Figure 3). The relative molecular weight (Mr) is slightly higher than the expected 22 kDa due to the presence of myc-tags in the PTP4A3v1 vector. Under the experimental conditions used, the endogenous level of PRL-3 protein expression in MCF-7 cells was below the limit of detection.

### Protein expression of PRL-3 in preinvasive and invasive breast cancer, and corresponding lymph node metastases

**Figure 4:**
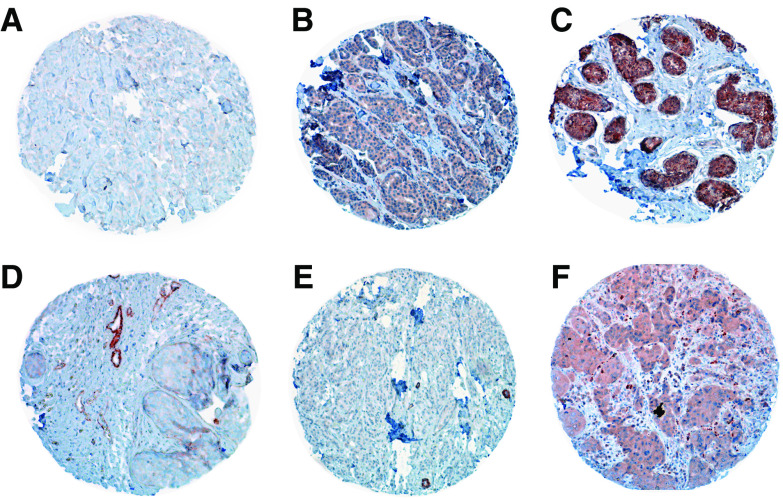
Invasive breast cancer specimens from the tissue microarray with (**A**) negative, (**B**) moderate, and (**C**) strong positive staining reaction for PRL-3. (**D**) Strong PLR-3 staining of tumour vessel. (**E**) PRL-3 negative and (**F**) positive lymph node metastases. Magnification 10-fold.

Expression of PRL-3 was analysed semiquantitatively by immunohistochemistry in 135 DCIS and 147 invasive breast carcinomas. In addition, a validation set of 99 invasive breast cancer samples was examined. PRL-3 immunoreactivity was mainly located in the cytoplasm, rarely also a nuclear staining was seen in addition. Moreover, strong PRL-3 staining intensity was observed in breast tumour vessels, but the stroma was always negative. 116 of 135 (85.9%) of DCIS showed a moderate to strong staining intensity and were therefore defined as ‘positive’. In invasive breast carcinomas, 111 of 147 (75.5%) and 79 out of 99 (79.8%) tumour specimens stained positive for PRL-3. From the 147 breast cancers studied in subset 1, 80 were ductal invasive breast cancers of which again 80% (*n*=64) were PRL-3 positive. Table 2 provides detailed information on gradual assessment of PRL-3 staining. 22 of 24 (91.7%) lymph node metastases showed a positive staining for PRL-3, whereas only 16 (66.7%) of the corresponding primary tumours were PRL-3 positive. This difference was statistically significant (*P*=0.033). Figure 4 shows samples of PRL-3-negative and -positive core specimens.

### Association of PRL-3 expression with clinicopathological variables

**Figure 5:**
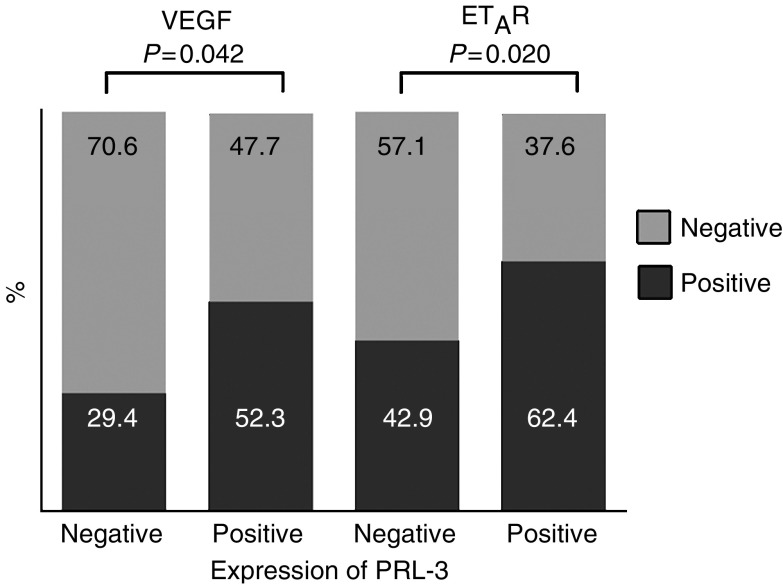
Expression of PRL-3 correlated with VEGF and ET_A_R expression, respectively.

In DCIS, there was no correlation of PRL-3 expression with nuclear grading. For invasive breast cancer no significant correlation between PRL-3 expression and common clinicopathological parameters such as tumour stage, nodal involvement, histologic grading, hormone receptor status etc. was observed in subset 1 (data not shown). In the series with longer follow-up (subset 2), patients with PRL-3 overexpressing tumours developed more frequently distant metastases (*χ*^2^ test, *P*=0.049). With respect to angiogenic factors, we observed a close positive correlation between PRL-3 expression and VEGF (*P*=0.042) as well as ET_A_R (*P*=0.020) expression in invasive breast carcinomas (Figure 5).

### Confocal immunofluorescence

**Figure 6:**
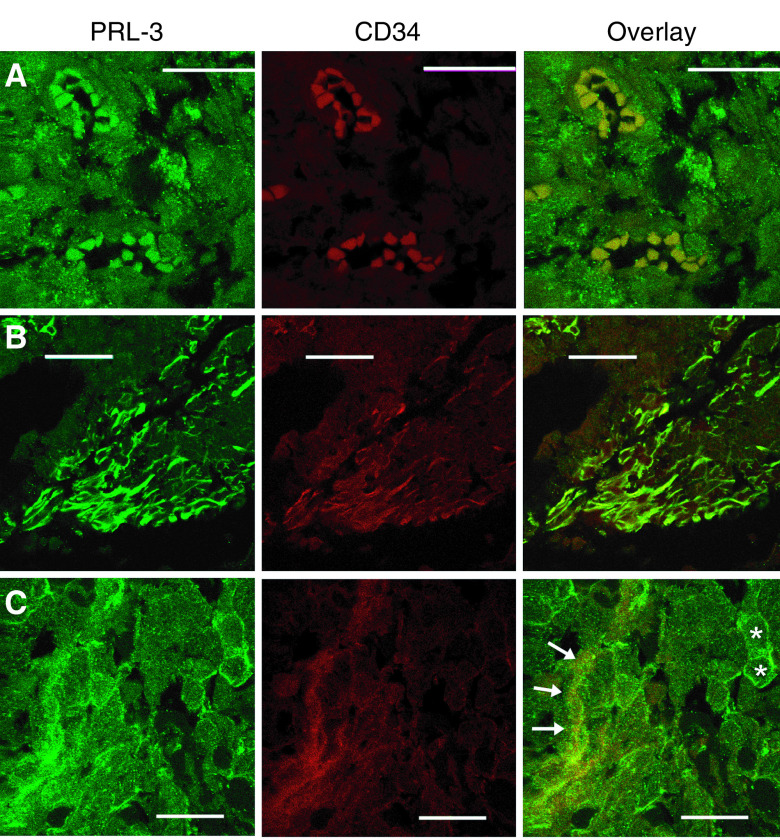
Colocalisation of PRL-3 with the vascular endothelial marker CD34 in breast cancer tissue. Left panels=PRL-3 staining (green fluorescent secondary antibody), central panels=CD34-staining (red fluorescent secondary antibody), right panels=merged image, yellow staining denotes colocalisation; (**A**) Colocalisation of PRL-3 with CD34-positive cells forming small blood vessels; (**B**) colocalisation of PRL-3 with CD34-positive blood vessels; (**C**) PRL-3 expression is not restricted to CD34-positive blood vessels (arrows) but occurs also in CD34-negative tumour cells (asterisks); bar=25 *μ*m.

Immunofluorescence colabeling of PRL-3 and the vascular endothelial marker CD34 was performed to proof expression of PRL-3 not only in breast cancer tumour cells, but also in tumour vasculature. Colocalisation of PRL-3 with CD34 in breast cancer tissue was evaluated using confocal laser immunofluorescence. PRL-3 showed colocalisation with CD34-positive cells and blood vessels, but was also expressed in CD34-negtative tumour cells, as shown in Figure 6.

### Prognostic value of PRL-3 expression in breast cancer

**Figure 7:**
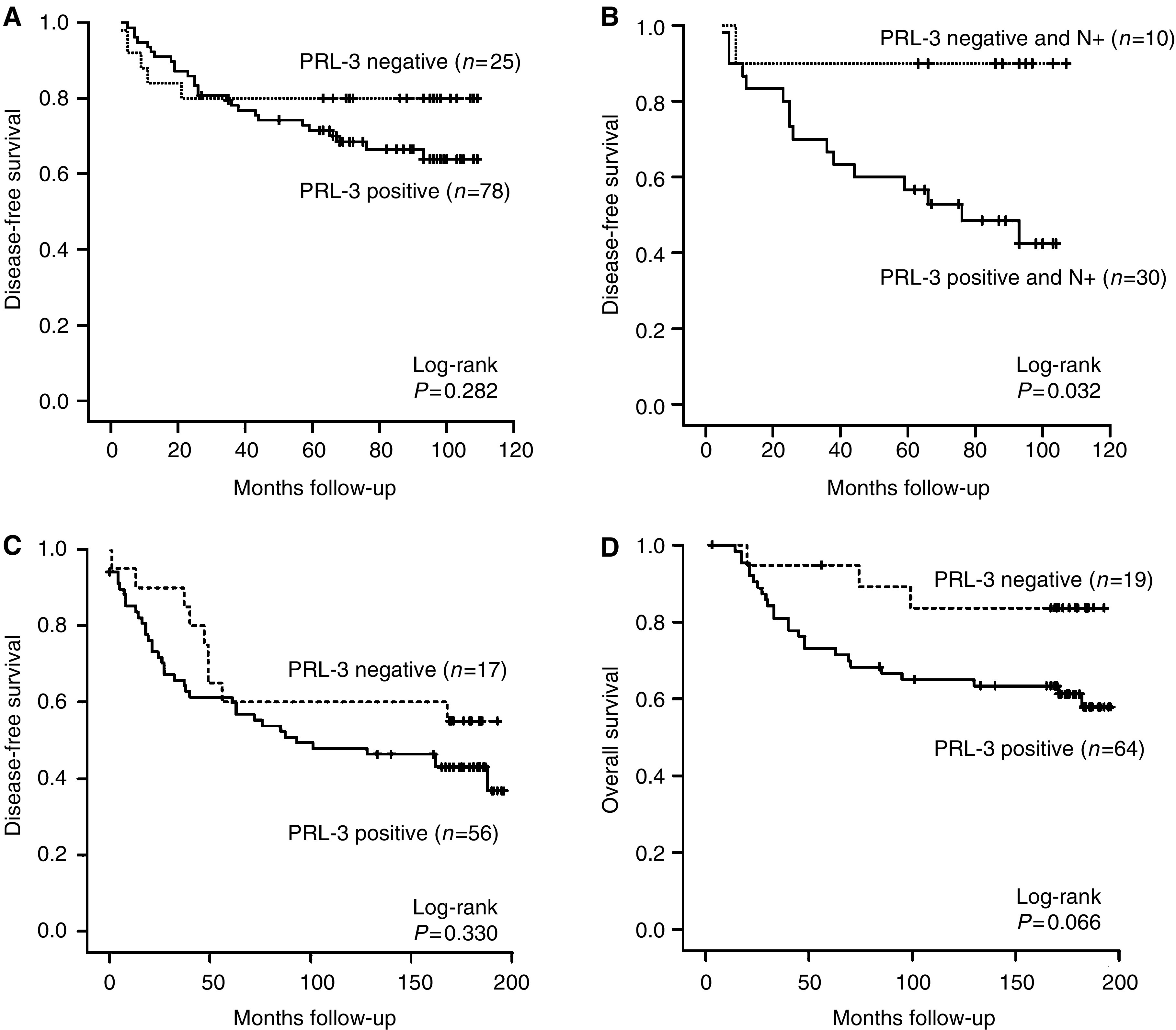
Kaplan–Meier estimates for DFS time and OS with respect to PRL-3 expression and clinicopathological parameters. (**A**) Disease-free survival time stratified by PRL-3 expression. (**B**) Survival curves for the subgroups of patients with nodal-positive invasive breast cancer stratified by PRL-3 expression. (**C**) Disease-free survival and (**D**) OS in subset 2, a set of patients with longer follow-up, stratified by PRL-3 expression.

Analysis of the association of PRL-3 expression with survival was performed for patients with invasive breast cancer. In subset 1, patients with PRL-3-positive breast carcinomas showed a trend towards shorter DFS compared to patients with PRL-3-negative cancers (83±4 months (95% CI, 74–91) *vs* 89±8 months (95% CI, 74–105), respectively). However, this difference was statistically not significant (*P*=0.282). However, in the group of node-positive breast cancer patients PRL-3 expression was associated with a significantly worse DFS (66±7 months (95% CI 52–80) compared to patients with PRL-3-negative tumours (97±9 months (95% CI 79–115); *P*=0.032). The corresponding Kaplan–Meier curves are shown in Figure 7. Interestingly, DFS in nodal-positive but PRL-3-negative patients is comparable to that of nodal-negative patients (97±9 months (95% CI, 79–115) and 94±10 months (95% CI, 75–113), respectively). No significant difference was observed between OS and PRL-3 expression.

Corresponding to our results from subset 1, a trend towards shorter DFS in PRL-3-positive carcinomas compared to patients with PRL-3-negative carcinomas was also observed the series of patients with longer follow-up (subset 2) with a mean DFS of 118±11 months (95% CI, 97–139) in PRL-3-positive *vs* 138±18 months (95% CI, 103–172; *P*=0.330) in PRL-3-negative patients, respectively. Since in this subset only three patients with lymph node involvement had PRL-3-negative tumours, a subgroup analysis of node-positive patients with respect to PRL-3 expression was not feasible. There was a trend towards shorter OS in PRL-3-positive patients in subset 2: 139±9 months (95% CI, 121–158) *vs* 172±11 months (95% CI 149–194; *P*=0.066), respectively.

## DISCUSSION

A number of emerging new prognostic markers of breast cancer have been investigated. The presence or absence of lymph node metastasis is still regarded as the most valuable single prognostic attribute (Goldhirsch *et al*, 2005). However, additional markers are needed to facilitate a risk-directed treatment tailored to individual patients.

The purpose of the present study was to determine whether the PTPs, in particular PRL-3, could provide additional prognostic value in breast cancer patients. The PRL-3 mRNA expression was significantly elevated in neoplastic compared to normal breast tissue. For PRL-2 and PRL-1 mRNA expression no significant differences were found. Therefore, at the protein level, we focused on PRL-3 expression in preinvasive and invasive breast cancer as well as in corresponding lymph node metastases using TMA.

PRL-3 protein expression was seen in 75.5% of all invasive breast cancers and 85.9% of DCIS. The higher expression rate in DCIS was somewhat surprising but has similarly been shown for HER2 expression: HER2 representing a marker of poor prognosis in breast cancer. It is involved in normal breast growth and development, and overexpression of HER2 seems to play a role in malignant transformation and tumourigenesis. HER2 was first reported in preinvasive breast cancer, and higher expression in DCIS than in IDC has been demonstrated (Ross and Fletcher, 1998; Menard *et al*, 2001). The explanation of this phenomenon was, that HER2-negative IDCs might not derive from DCIS, but develop from another lesion, the atypical ductal hyperplasia (ADH), which showed no HER2 amplification (Menard *et al*, 2001). The same might be the case in PRL-3 expression, but to date, there is no data on the expression of PRL-3 in ADH.

PRL-3 overexpression has been reported for colorectal, liver, and gastric cancer (Stephens *et al*, 2005) and recently for ovarian cancer (Polato *et al*, 2005). PRL-3 has been shown to influence proliferation, migration, and metastasis of cancer cells *in vitro* and *in vivo* (Cates *et al*, 1996; Wang *et al*, 2002; Zeng *et al*, 2003). Several studies have demonstrated the relevance of tumour angiogenesis for these particular tumour cell properties (Folkman and Klagsbrun, 1987; Hanahan and Weinberg, 2000; Alberts, 2002; Ambler *et al*, 2003). In a xenograft model PRL-3 expressing tumours showed a dense formation of tubular structures with histological similarity to blood vessels. Also, these PRL-3 overexpressing tumours were highly vascularised. In contrast, the PRL-3-negative controls did not show these features (Guo *et al*, 2004). Therefore, PRL-3 might play a causative role in tumour-related angiogenesis. Consistently, for breast and CRC PRL-3 overexpression has been described especially in the tumours endothelial cells (Bardelli *et al*, 2003; Parker *et al*, 2004). Vascular endothelial growth factor is known to be another important factor for tumour angiogenesis. Vascular endothelial growth factor has been shown to stimulate the migration of endothelial cells, the formation of blood vessels in tumours (Ambler *et al*, 2003) and is associated with an adverse outcome of breast cancer patients (Gasparini, 2001). In this study, we found a positive correlation between PRL-3 and VEGF expression in breast cancer cells. Moreover, we observed a positive association of PRL-3 expression with expression of the ET_A_R, another proangiogenic factor in breast cancer (Nelson *et al*, 2003; Wülfing *et al*, 2004). To strengthen the hypothesis that PRL-3 plays a role in tumour angiogenesis we performed confocal immunofluorescence of PRL-3 and CD34, a specific marker for endothelial cells, widely used in microscopic evaluation of tumour angiogenesis (reviewed in McDonald and Choyke, 2003). We could demonstrate that PRL-3 is expressed in CD34-positive endothelial cells and microvessels as well as in CD34-negative tumour cells. These findings suggest that PRL-3 expression may also play an autocrine and paracrine role in breast cancer angiogenesis.

PRL-3 protein expression previously has been analysed in other adenocarcinomas, in particular in colorectal and gastric cancer. For CRC higher PRL-3 gene and protein expression in metastases than in nonmetastatic tumours and normal colorectal epithelium were reported (Peng *et al*, 2004). In gastric, colorectal and ovarian cancer, PRL-3 expression was associated with tumour stage and extent of lymph node metastasis (Miskad *et al*, 2004), and for CRC a negative prognostic impact with shorter survival was found (Peng *et al*, 2004). *In vitro*, knockdown of PRL-3 in ovarian cancer cell lines with small interfering RNA resulted in impaired cancer cell growth (Polato *et al*, 2005).

In this study, we also analysed expression of PRL-3 in breast carcinomas and their corresponding lymph node metastases. As shown for other tumour entities, we have found a significantly more frequent expression of PRL-3 in lymph node metastases as compared to the corresponding primary tumour. Moreover, we observed a higher incidence of subsequent distant metastases in patients with PRL-3 overexpressing breast carcinomas. In addition, Rouleau *et al* (2006) could demonstrate, that PRL-3 actively promotes invasiveness of MCF-7 cells *in vitro*. In summary, overexpression of PRL-3 might facilitate tumour cells to invade into lymphatics or vasculature, and therefore being a prerequisite for development of local lymph node and distant metastases.

PRL-3 expression seems to adversely influence DFS in breast cancer patients. We found PRL-3 expression to correlate with decreased DFS. This negative prognostic impact was in particular pronounced in lymph node-positive breast cancer patients. Conversely, patients with PRL-3-negative tumours showed a significantly longer DFS. Absence of PRL-3 expression even abrogated the survival difference between node-negative and node-positive patients. Taking into account that the presence or absence of lymph node metastases is regarded the most valuable single prognostic factor, these results are interesting. The relatively small set of PRL-3-negative tumours in our study (24.5%) might represent a group of patients with unexpectedly good prognosis, even in case of lymph node involvement. In patients with breast cancer, a long observational period is necessary to discriminate patients with respect to prognosis. The trend towards an impaired DFS observed in our pilot study was confirmed in a subsequent study performed for validation of these findings. This validation set was characterised by a longer period of follow-up as compared to the initial subset. In this subset, we found a trend towards a shorter OS in PRL-3-positive breast cancer patients. The longer follow-up period in this subset might explain why effects of PRL-3 expression on OS became detectable only in this subset of patients. There was no correlation between PRL-3 expression and conventional clinicopathological prognostic markers.

In view of our findings expression of PRL-3 may facilitate identification of patients who are at high risk for disease recurrence (PRL-3-positive tumours) or conversely of patients who have an unanticipated good prognosis despite lymph node metastases (PRL-3-negative tumours, respectively). Adjuvant systemic therapy has been proven to reduce mortality from breast cancer (EBCTCG, 2005). However, some patients receive therapy with little or no likelihood of benefit. For example, patients with early stage, lymph node-negative breast cancer have approximately 25% risk of dying from breast cancer within 10 years if they do not receive adjuvant treatment, whereas 75% however, will survive 10 years even without any further treatment (Chia *et al*, 2004). Therefore, reliable prognostic markers are needed to help selecting those patients who most likely benefit from systemic therapy and need even more intensive treatment because of high risk of disease recurrence or death. Further studies are necessary to confirm that the analysis of PRL-3 expression in breast cancer might help select patients who are at higher risk for disease recurrence and therefore should receive appropriate systemic treatment.

Moreover, PRL-3 might be a new therapeutic target. Pentamidine, an antileishmaniasis drug with unknown mechanism of action has been shown to inhibit PRL phosphatases *in vitro* and may provide a basis for developing novel PTPase-targeted therapeutics (Pathak *et al*, 2002). Also, farnesyltranferase inhibitors are suspected to function not only through inhibition of members of the Ras oncogene family, but as well through inhibition of PRL-PTPs (Sebti and Der, 2003).

In summary, we have shown that PRL-3 is expressed in breast cancer. PRL-3 expression seems to adversely influence disease outcome, being related to a shorter DFS in breast cancer patients. Our findings suggest that analysis of PRL-3 expression might serve as an additional, prognostic factor in breast cancer and could be useful for choice of risk-adapted, more tailored treatment concepts for the individual patient.

## Figures and Tables

**Table 1:** Distribution of TMN^a^ stages and histological grade in the reported series of breast cancer patients (*n*=147 in subset 1 and *n*=99 in subset 2)

**Clinicopathological parameter**	**Subset 1 *n* (%)**	**Subset 2 *n* (%)**
*Tumour stage* b		
pT1	52 (35.6%)	37 (37.4%)
pT2	54 (37%)	43 (43.4%)
pT3	12 (8.2%)	8 (8.1%)
pT4	28 (19.2%)	10 (10.1%)
*Lymph nodes* b		
pN0	75 (53.6%)	56 (56.6%)
pN1	53 (37.9%)	25 (25.3%)
pN2	11 (7.9%)	18 (18.2%)
pN3	1 (0.7%)	0 (0%)
*Metastasis at diagnosis* c		
No	129 (87.8%)	80 (95.2%)
Yes	18 (12.2%)	4 (4.8%)
*Histological grade* c		
G1	14 (9.5%)	15 (15.3%)
G2	75 (51%)	64 (65.3%)
G3	58 (39.5%)	19 (19.4%)

a TNM, Tumour-Node-Metastasis.

b Information on pT stage was available in 146 of 147 (99.3%), on pN stage in 140 of 147 (95.9%) patients in subset 1.

c In subset 2, information on pM stage was only available in 84 of 99 (84.8%), on histological grading in 98 of 99 (99.0%) patients.

**Table 2:** Immunohistochemical analysis of PRL-3 expression in DCIS and invasive breast cancer

	**DCIS**	**Invasive breast cancer**	
**Score**	***n (%)***	**Subset 1 *n (%)***	**Subset 2 *n (%)***
0	19 (14.1)	36 (24.5)	20 (20.2)
1+	60 (44.4)	74 (50.3)	63 (63.6)
2+	56 (41.5)	37 (25.2)	16 (16.2)
Negative (score 0)	19 (14.1)	36 (24.5)	20 (20.2)
Positive (score 1–2)	116 (85.9)	111 (75.5)	79 (79.8)

DCIS, ductal breast carcinoma *in situ*.
